# A randomised, single-blind, placebo-controlled, dose-finding safety and tolerability study of the anti-CD3 monoclonal antibody otelixizumab in new-onset type 1 diabetes

**DOI:** 10.1007/s00125-020-05317-y

**Published:** 2020-11-04

**Authors:** Bart Keymeulen, André van Maurik, Dave Inman, João Oliveira, Rene McLaughlin, Rachel M. Gittelman, Bart O. Roep, Pieter Gillard, Robert Hilbrands, Frans Gorus, Chantal Mathieu, Ursule Van de Velde, Nicolas Wisniacki, Antonella Napolitano

**Affiliations:** 1 Academic Hospital and Diabetes Research Center, Vrije Universiteit Brussel, Brussels, Belgium; 2 Belgian Diabetes Registry, Brussels, Belgium; 3 GlaxoSmithKline Medicines Research Centre, Stevenage, UK; 4 GlaxoSmithKline, Global Clinical Operations, Cambridge, UK; 5 Department of Immunology and Blood Transfusion, Leiden University Medical Center, Leiden, the Netherlands; 6 Adaptive Biotechnologies, Seattle, WA USA; 7 Department of Internal Medicine, Leiden University Medical Center, Leiden, the Netherlands; 8 Department of Diabetes Immunology, Diabetes & Metabolism Research Institute, Beckman Research Institute at the City of Hope, Duarte, CA USA; 9 Department of Endocrinology, University Hospitals Leuven-KUL, Leuven, Belgium

**Keywords:** Anti-CD3 monoclonal antibody, Autoreactive T cell, Epstein–Barr virus reactivation, Islet autoimmunity, Type 1 diabetes

## Abstract

**Aims/hypothesis:**

Numerous clinical studies have investigated the anti-CD3ɛ monoclonal antibody otelixizumab in individuals with type 1 diabetes, but limited progress has been made in identifying the optimal clinical dose with acceptable tolerability and safety. The aim of this study was to evaluate the association between dose–response, safety and tolerability, beta cell function preservation and the immunological effects of otelixizumab in new-onset type 1 diabetes.

**Methods:**

In this randomised, single-blind, placebo-controlled, 24 month study, conducted in five centres in Belgium via the Belgian Diabetes Registry, participants (16–27 years old, <32 days from diagnosis of type 1 diabetes) were scheduled to receive placebo or otelixizumab in one of four dose cohorts (cumulative i.v. dose 9, 18, 27 or 36 mg over 6 days; planned *n* = 40). Randomisation to treatment was by a central computer system; only participants and bedside study personnel were blinded to study treatment. The co-primary endpoints were the incidence of adverse events, the rate of Epstein–Barr virus (EBV) reactivation, and laboratory measures and vital signs. A mixed-meal tolerance test was used to assess beta cell function; exploratory biomarkers were used to measure T cell responses.

**Results:**

Thirty participants were randomised/28 were analysed (placebo, *n* = 6/5; otelixizumab 9 mg, *n* = 9/8; otelixizumab 18 mg, *n* = 8/8; otelixizumab 27 mg, *n* = 7/7; otelixizumab 36 mg, *n* = 0). Dosing was stopped at otelixizumab 27 mg as the predefined EBV reactivation stopping criteria were met. Adverse event frequency and severity were dose dependent; all participants on otelixizumab experienced at least one adverse event related to cytokine release syndrome during the dosing period. EBV reactivation (otelixizumab 9 mg, *n* = 2/9; 18 mg, *n* = 4/8: 27 mg, *n* = 5/7) and clinical manifestations (otelixizumab 9 mg, *n* = 0/9; 18 mg, *n* = 1/8; 27 mg, *n* = 3/7) were rapid, dose dependent and transient, and were associated with increased productive T cell clonality that diminished over time. Change from baseline mixed-meal tolerance test C-peptide weighted mean AUC_0–120_ min following otelixizumab 9 mg was above baseline for up to 18 months (difference from placebo 0.39 [95% CI 0.06, 0.72]; *p* = 0.023); no beta cell function preservation was observed at otelixizumab 18 and 27 mg.

**Conclusions/interpretation:**

A metabolic response was observed with otelixizumab 9 mg, while doses higher than 18 mg increased the risk of unwanted clinical EBV reactivation. Although otelixizumab can temporarily compromise immunocompetence, allowing EBV to reactivate, the effect is dose dependent and transient, as evidenced by a rapid emergence of EBV-specific T cells preceding long-term control over EBV reactivation.

**Trial registration:**

ClinicalTrials.gov NCT02000817.

**Funding:**

The study was funded by GlaxoSmithKline.

**Figure:**
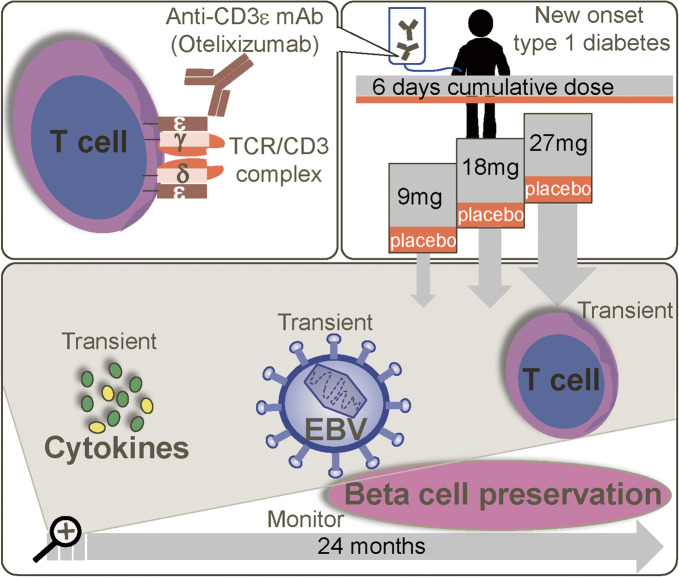
Graphical abstract

**Supplementary Information:**

The online version of this article (10.1007/s00125-020-05317-y) contains peer-reviewed but unedited supplementary material, which is available to authorised users.

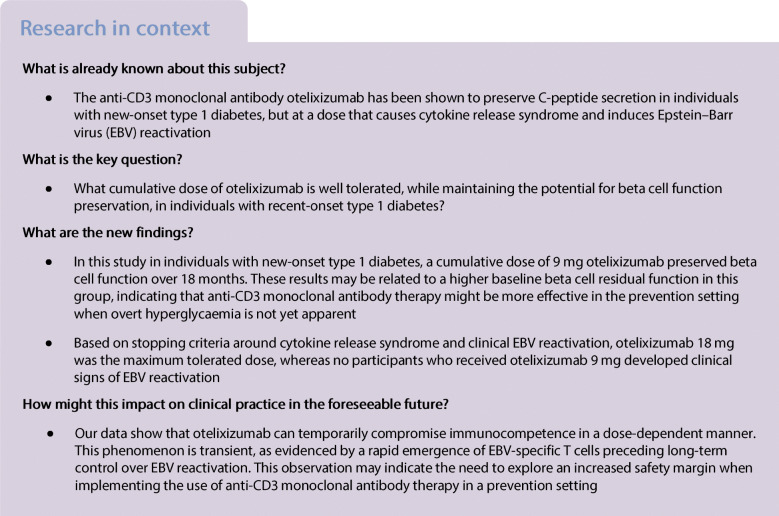



## Introduction

Type 1 diabetes is a chronic, progressive disease characterised by T cell-mediated autoimmune destruction of insulin-producing pancreatic beta cells [1, 2]. The cytotoxic T cell infiltrate in inflamed islets is dominated by CD8^+^ lymphocytes, but may also contain CD4^+^ lymphocytes, B lymphocytes and macrophages [3]. Clinical symptoms usually develop when most functional beta cell mass has been lost [4], by which time individuals with type 1 diabetes depend on exogenous insulin and lifestyle management as the mainstays of treatment [5]. Even with intensive insulin therapy, individuals with type 1 diabetes are at risk of acute complications such as hypoglycaemia and ketoacidosis, and of long-term microvascular and macrovascular complications [6, 7].

Therapies targeting T cells have been an area of much interest in new-onset type 1 diabetes. In particular, monoclonal antibodies against CD3 can change the natural course of type 1 diabetes, leading to a longer preservation of beta cell function [8]. High cumulative doses of humanised anti-CD3 antibodies have been shown to maintain baseline plasma C-peptide levels over at least 18 months in a subgroup of individuals with stage 3, recent-onset type 1 diabetes [9–13]; however, clinically relevant metabolic effects have to be weighed against side effects during and after treatment, as anti-CD3 antibody administration can cause reactivation of Epstein–Barr virus (EBV) and induce a local (if subcutaneously administered [14]) or systemic (if intravenously administered [9, 10]) cytokine release response.

Otelixizumab is a humanised, Fc-modified, non-mitogenic, anti-CD3 monoclonal antibody with limited Fc receptor binding [9, 15]. A Phase II, placebo-controlled trial of otelixizumab (total dose of 48 mg administered over 6 days) demonstrated preserved residual beta cell function for at least 18 months in participants with recent-onset type 1 diabetes; however, 75% of those treated with otelixizumab developed symptoms consistent with acute mononucleosis, and all participants who received otelixizumab had transient adverse events (AEs) associated with cytokine release syndrome (CRS) [9]. In the Phase III Durable Response Therapy Evaluation for Early or New-Onset Type 1 Diabetes studies, which evaluated a total dose of otelixizumab of 3.1 mg (administered over 8 days), treatment was well tolerated but was not efficacious in terms of beta cell preservation [16, 17], highlighting the need to establish an optimal otelixizumab dose in terms of both safety and efficacy.

The primary aim of this Phase I/II study was to identify a dosage regimen of intravenously administered, escalating doses of otelixizumab with an acceptable safety and tolerability profile, which is considered a necessary first step to determining the therapeutic index of otelixizumab. In addition, the C-peptide rate of decline in participants with new-onset type 1 diabetes and detailed immunological mechanisms were investigated. To this end, partially exhausted CD8^+^ memory T cells were monitored to evaluate any association with metabolic response in halting or slowing β-cell destruction in this population. Furthermore, changes in the T cell repertoire and EBV-reactive CD8^+^ T cells were investigated to gain insights into immunological responses following EBV reactivation. The pharmacological findings from this study have been reported previously, and showed that serum otelixizumab concentrations increased with ascending doses (9, 18 and 27 mg) and CD3ɛ target engagement was rapidly achieved with all doses, with the two highest doses achieving approximately 90% target engagement and consequential CD3ɛ/T cell receptor (TCR) downmodulation by day 6 [18].

## Methods

### Study design and study population

Full details of the study design, study population and ethics approval have been reported elsewhere [18] and are described in brief here. This study was a randomised, single-blind, placebo-controlled, dose-ascending study, conducted in five centres in Belgium via the Belgian Diabetes Registry between March 2014 and September 2018 (ClinicalTrials.gov NCT02000817). Otelixizumab was planned to be dosed by daily i.v. infusions over 6 days in four dose cohorts at a total dose of 9 mg (cohort 1), 18 mg (cohort 2), 27 mg (cohort 3) or 36 mg (cohort 4). Participants were randomised to treatment by a secure, central computer system using a randomisation schedule generated by GlaxoSmithKline Clinical Statistics, UK. Only participants and bedside study personnel were blinded to study treatment. In addition, the sponsor team members were unblinded to study treatment. Participants were dosed in hospital for the first 3 days (slower rate of infusion [17]) and as outpatients on days 4–6; the otelixizumab dose was divided equally over the 6 days of dosing. Full details of the dosing regimens are shown in electronic supplementary material (ESM) Table 1. Following the last day of dosing, participants were followed up as outpatients at days 14 and 21, weeks 4 and 6, and months 2, 3, 6, 9, 12, 18 and 24. Prophylaxis for CRS included non-steroidal anti-inflammatory drugs and non-sedating oral antihistamines (cetirizine) and was given to all participants 1–2 h prior to and during dosing with otelixizumab. In addition, the antiemetic ondansetron, a 5HT_3_-receptor antagonist, was permitted for those receiving otelixizumab 18 mg or more (as per protocol amendment).

Individuals were eligible for the trial if they were 16–27 years old, less than 32 days from a diagnosis of type 1a (autoimmune) diabetes and positive for at least one autoantibody associated with type 1 diabetes, and had evidence of residual functioning beta cells as measured by a mixed-meal stimulated C-peptide peak level of ≥0.2 nmol/l. Participants who were positive for EBV capsid antibody IgM in the absence of positive EBV nuclear antigen IgG, had an EBV viral load of >10,000 copies/10^6^ peripheral blood mononuclear cells (PBMCs) or who were IgG negative for EBV were excluded.

Dose escalation (i.e. initiation of treatment in next participant cohort) was decided upon pre-established stopping criteria around in-stream, real-time data review of CRS variables based on the maximum number of participants experiencing CRS symptoms of grade 3 or worse (three out of eight participants on active treatment) (ESM Table 2) and the number of participants showing clinical and virological EBV reactivation (Table 1).

**Table 1:** Protocol-defined dose-escalation EBV reactivation stopping criteria

EBV reactivation	Criteria^a^
Clinical symptoms of mononucleosis	Fever, fatigue, malaise, myalgia, pharyngodynia, lymphadenopathy
Change in viral load	EBV by PCR >10,000 copies/10^6^ PBMCs
Change in serology	Emergence of IgM for EBV on otherwise EBV IgG-positive participants

^a^Stopping criteria: If three or more of eight participants on active treatment within a cohort developed symptoms of clinical EBV reactivation, otelixizumab dosing was stopped and no further dosing was carried out on any other participants; participants who had been dosed were followed up as per protocol

The study was carried out with the approval of the ethics committees of all participating hospitals, the Belgian Diabetes Registry and the Belgian national regulatory agency. Written consent was obtained from all participants older than 18 years, and from participants aged 16–18 years and their parents/guardians.

### Safety

The primary study objective was to assess the safety and tolerability of otelixizumab over 24 months. Safety assessments, including clinical laboratory tests (haematology, clinical chemistry and serology), vital signs, ECGs and physical examinations were conducted every day during the dosing period and at regular intervals during the 24 month follow-up. Specific liver chemistry stopping criteria were followed as detailed in the ESM Methods. Liver function variables were assayed by a local laboratory as part of routine clinical chemistry laboratory testing. AEs were monitored throughout the study.

#### EBV viral load detection

Blood samples for EBV viral load detection were collected at predose baseline, predose on day 6 of dosing, at day 21, and at months 2, 3, 6 and 24. EBV load was quantified per 10^6^ PBMCs. During the life-phase of the study, measurement of EBV DNA load was performed on DNA extracted from PBMCs using a quantitative real-time PCR assay (Abbott RealTime EBV assay; Abbott Molecular, USA). The assay was validated and performed by Q^2^ Solutions (UK) [19].

### Efficacy

#### Metabolic endpoints

A mixed-meal tolerance test (MMTT) was conducted at months 3, 6, 12, 18 and 24 to determine the blood levels of C-peptide and glucose in response to a standardised amount of Ensure powder (Abbott, the Netherlands). Blood samples were collected at 10 min before, immediately before and 15, 30, 60, 90 and 120 min after meal consumption. The MMTT C-peptide AUC from time 0 to 120 min (AUC_0–120_ min) was calculated using the trapezoidal rule, normalised for time interval. A metabolic responder was defined as a participant with a change in C-peptide MMTT weighted mean AUC at 24 months of <40% from baseline [20].

A hyperglycaemic clamp procedure [9] was performed at baseline, and after 6 and 24 months. Plasma C-peptide and glucose levels were measured during the procedure at: 150, 165 and 180 min during the euglycaemic low phase and at 0, 60, 90, 120 and 140 min during the hyperglycaemic high phase. C-peptide AUC from time 60 to 140 min during the hyperglycaemic high phase was calculated and expressed as value per min for each period. The insulin sensitivity index was calculated [21]. The MMTT and hyperglycaemic clamp tests were separated by at least 4 days.

Mean daily insulin use (IU [kg body weight]^−1^ day^−1^) was calculated over the 7 days prebaseline, on days 14 and 21, at weeks 4 and 6, and at months 2, 3, 6, 9, 12, 18 and 24. Measurement of HbA_1c_ was performed at predose baseline and months 6 and 24 [22].

### Immunogenicity

Blood samples to assess serum anti-drug antibody levels were collected at baseline and months 3 and 6. Samples were analysed using a validated immuno-electrochemiluminescence assay (Drug Metabolism and Pharmacokinetics, GlaxoSmithKline, USA).

### Exploratory biomarkers

#### Flow cytometry

Lymphocyte subsets were phenotyped and quantified by flow cytometry, as detailed in ESM Methods.

#### TCR immunosequencing

T cell clonality was determined using the TCRB immunoSEQ Assay (Adaptive Biotechnologies, USA), as detailed in ESM Methods.

### Statistical analysis

Sample sizes were based on feasibility, to primarily allow characterisation of the safety and tolerability of ascending doses of otelixizumab in individuals with type 1 diabetes. A total of 40 participants were planned for the study, comprising eight participants randomised to otelixizumab and two to placebo in each cohort. All safety data are summarised descriptively. Change from baseline in C-peptide and glucose levels following the MMTT and hyperglycaemic clamp tests were analysed using a mixed-model repeated-measures analysis adjusted for treatment, visit and baseline. Non-parametric statistical tests were used to compare the level of productive clonality between the placebo and active-treatment groups (Mann–Whitney *U* test) and productive clonality over time in each treatment group (Wilcoxon signed-rank test).

## Results

### Participant characteristics

**Fig. 1:**
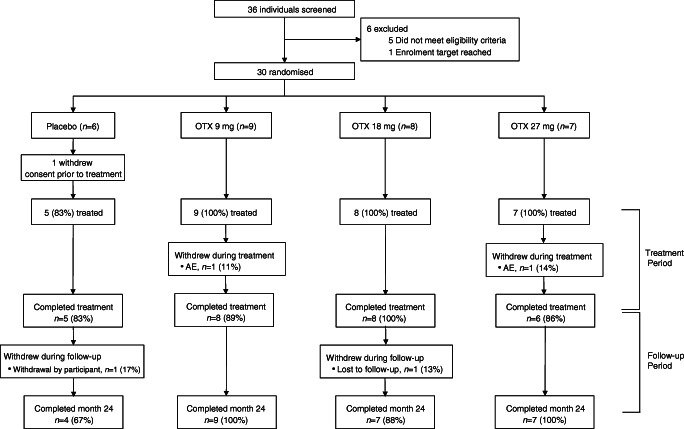
Participant flow diagram. OTX, otelixizumab

Of 36 individuals screened, 30 were randomised to treatment: six with placebo (two in each of cohorts 1, 2 and 3), nine with otelixizumab 9 mg (cohort 1), eight with otelixizumab 18 mg (cohort 2) and seven with otelixizumab 27 mg (cohort 3) (Fig. 1). No participants were randomised to receive otelixizumab 36 mg (cohort 4) as the predefined stopping criteria on EBV reactivation were met at the 27 mg dose. Two participants withdrew from otelixizumab treatment: one in the 9 mg group because of an AE on day 2 of treatment and one in the 27 mg group because of a serious AE (SAE) on day 5 of treatment. Both participants remained in the trial and completed a minimum of 24 months of safety follow-up. Three participants withdrew from the study or were lost to follow-up: two participants in the placebo group withdrew themselves (one prior to receiving any treatment and one during follow-up); and one participant in the 18 mg group was lost to follow-up. All data collected from participants who withdrew from the treatment or study were included in the relevant analyses up to the point of study withdrawal.

Baseline characteristics were generally similar across the groups (Table 2).

**Table 2:** Baseline characteristics

Characteristic	Placebo (*n* = 5)	OTX 9 mg (*n* = 9)	OTX 18 mg (*n* = 8)	OTX 27 mg (*n* = 7)
Age, years	24.8 ± 4.44	22.4 ± 2.13	20.5 ± 4.31	22.1 ± 3.98
Age group 16–17 years, *n*	1	0	3	1
Age group ≥18 years, *n*	4	9	5	6
Male/female, *n*	3/2	6/3	5/3	5/2
BMI, kg/m^2^	22.7 ± 2.12	23.1 ± 4.28	21.4 ± 2.01	21.4 ± 2.21
Height, cm	169.2 ± 8.66	177.0 ± 8.95	172.8 ± 9.73	174.4 ± 7.50
Weight, kg	65.2 ± 10.61	72.8 ± 16.12	64.2 ± 9.55	65.3 ± 8.71
Race				
White	5 (100)	9 (100)	8 (100)	7 (100)
Autoantibody prevalence at screening				
Multiple autoantibody positive	5 (100)	9 (100)	7 (88)	7 (100)
GADA positive	5 (100)	6 (67)	8 (100)	7 (100)
IA2A positive	4 (80)	7 (78)	7 (88)	6 (86)
Znt8A positive	3 (60)	8 (89)	2 (25)	5 (71)
IAA positive	1 (20)	3 (33)	2 (25)	1 (14)
HbA_1c_, %	8.1 ± 1.74	7.1 ± 1.53	6.8 ± 1.48	6.8 ± 1.80
HbA_1c_, mmol/mol	80.6 ± 17.40	70.8 ± 15.27	67.6 ± 14.79	68.1 ± 17.98
Insulin use in the 7 days before baseline, IU kg^−1^ day^−1^	0.46 ± 0.112	0.40 ± 0.210	0.37 ± 0.215	0.40 ± 0.235
Insulin sensitivity index (μmol kg^−1^ min^−1^ (pmol/l)^−1^)	0.0018 ± 0.00095	0.0015 ± 0.00076	0.0018 ± 0.00087	0.0015 ± 0.00114

Data are means ± SD or *n* (%), unless otherwise stated

Baseline refers to screening or day 1 prior to randomisation

GADA, GAD antibody; IAA, insulin autoantibody; IA2A, insulinoma-associated protein 2 autoantibody; OTX, otelixizumab; Znt8A, zinc transporter 8 autoantibody

### Safety

Overall, the frequency and severity of AEs were dose dependent, with a higher frequency in participants receiving otelixizumab (vs placebo) around the time of dosing (Table 3). During the dosing period, drug-related grade 2 and 3 AEs occurred with all otelixizumab doses and in similar percentages of participants (ESM Table 3). The most common AEs reported during the dosing period were headache, nausea, vomiting and rash (Table 3). Headache occurred in all participants on active treatment. Nausea and vomiting occurred less frequently in the otelixizumab 9 mg group (56% and 44%, respectively) compared with the 18 mg group (88% and 75%, respectively) and 27 mg group (both 100%). Rash occurred in active-treatment groups only, but not in a dose-dependent manner (otelixizumab 9 mg, 44%; 18 mg, 63%; 27 mg, 43%). One individual in the 9 mg group on active treatment stopped participation before the end of dosing because of CRS-related AEs (which did not meet the protocol stopping criteria), and one participant in the 27 mg group on active treatment was diagnosed with cytomegalovirus primo-infection on day 5 of the dosing period, reported as a drug-related SAE. This participant was not given the last dose of otelixizumab (day 6) and was immediately treated as per standard of care (ganciclovir). The infection and all symptoms resolved within 13 days. This SAE was reported as a suspected unexpected serious adverse reaction. The incidence of other SAEs during the 2 year follow-up was low (placebo, *n* = 1; otelixizumab 9 mg, *n* = 2; otelixizumab 18 mg, *n* = 1). None of these SAEs was assessed as drug-related.

**Table 3:** Summary of AEs

Type of AE	Placebo (*n* = 5)	OTX 9 mg (*n* = 9)	OTX 18 mg (*n* = 8)	OTX 27 mg (*n* = 7)
During dosing				
AEs related to study treatment	4 (80)	9 (100)	8 (100)	7 (100)
AEs leading to permanent discontinuation of study treatment	0	1 (11)	0	1 (14)
SAEs related to study treatment	0	0	0	1 (14)
Most frequent AEs related to study treatment^a^				
Headache	1 (20)	9 (100)	8 (100)	7 (100)
Nausea	1 (20)	5 (56)	7 (88)	7 (100)
Vomiting	0	4 (44)	6 (75)	7 (100)
Rash	0	4 (44)	5 (63)	3 (43)
Postdose to week 6				
AEs related to study treatment	3 (60)	7 (78)	7 (88)	7 (100)
Most frequent AEs related to study treatment^a^				
Rash	0	0	3 (38)	3 (43)
Skin exfoliation	0	3 (33)	0	0
Lymphadenopathy	0	0	1 (13)	3 (43)
Postdose week 6–month 24				
AEs related to study treatment^b^	5 (100)	8 (89)	5 (63)	5 (71)

Data are *n* (%)

^a^Excludes AEs related to the underlying condition (i.e. hypoglycaemia and hyperglycaemia)

^b^No events (other than hypoglycaemia and hyperglycaemia) occurred in more than one participant

OTX, otelixizumab

During the postdose to week 6 period, the most commonly reported AE assessed as drug-related (excluding those related to the underlying disease) was rash, which was observed to be dose related (Table 3). In this period, drug-related symptoms of grade 2 or higher also appeared to be dose dependent; no participants in the otelixizumab 9 mg group had postdose drug-related AEs of grade 2 or worse (ESM Table 3). Beyond Week 6, no patterns were observed in AEs related to study treatment, and there were no differences between active treatment and placebo.

At all doses, some transient increased liver function test results were observed during the dosing period, but none reached the protocol-defined stopping criteria. No significant abnormal clinical chemistry or haematology results were reported, apart from lymphocytopenia observed after day 1, which resolved at day 14 postdosing. No clinically significant changes in vital signs or ECGs were noted at any time point.

#### EBV reactivation

**Fig. 2:**
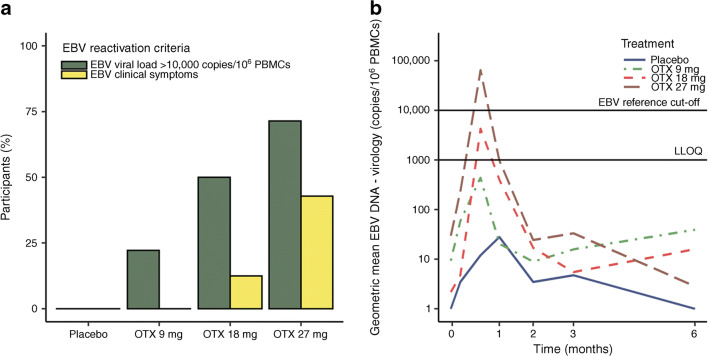
EBV reactivation showing: (**a**) proportion of participants with EBV viral load >10,000 copies/10^6^ PBMCs and proportion with EBV clinical symptoms; and (**b**) geometric mean copies of EBV viral load per 10^6^ PBMCs. LLOQ, lower limit of quantification; OTX, otelixizumab

A progressively higher number of participants had transient abnormally high EBV PCR levels with ascending otelixizumab dose, with peak levels measured at day 21 (Fig. 2). EBV reactivation and associated clinical signs were rapid, dose dependent and transient. EBV reactivation occurred in: otelixizumab (9 mg), *n* = 2/9; (18 mg), *n* = 4/8: (27 mg), *n* = 5/7 (Fig. 2). In the otelixizumab 18 and 27 mg groups, respectively, one out of eight and three out of seven participants had clinical signs of EBV reactivation (Fig. 2). No participants in the otelixizumab 9 mg group showed any clinical signs of EBV reactivation, but there was evidence of an increased EBV viral load in this group (Fig. 2).

### Efficacy

#### Metabolic endpoints

**Fig. 3:**
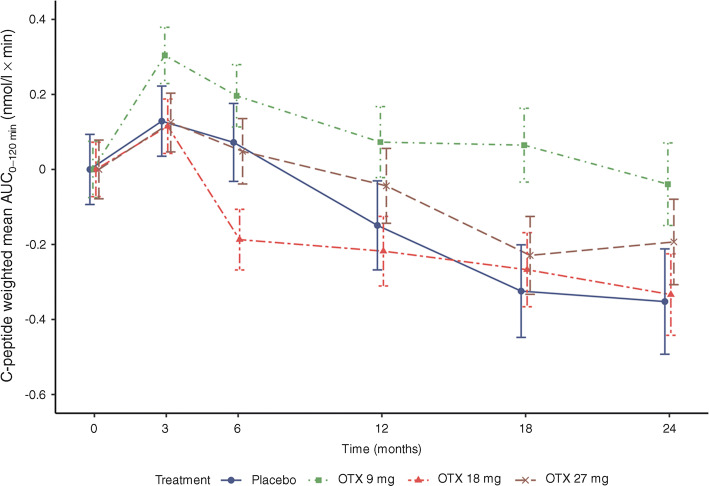
Change from baseline in LS mean (± SEM) of C-peptide weighted mean AUC_0–120 min_ following MMTT. LS: Least squares; OTX, otelixizumab

Following MMTT, the weighted mean AUC_0–120 min_ C-peptide (nmol/l × min) increased above baseline at 3 months and then progressively decreased over time in all groups (Fig. 3). In the otelixizumab 9 mg group, the change from baseline at month 18 was statistically significant compared with placebo (least-squares mean difference from placebo 0.39 [95% CI 0.06, 0.72], *p* = 0.023). In the other otelixizumab groups, mean AUC C-peptide was lower at month 18 than before treatment. Baseline mean C-peptide was numerically higher in the otelixizumab 9 mg group than in the other groups (baseline C-peptide weighted mean ± SD AUC_0–120_ min (nmol/l × min): placebo, 0.55 ± 0.25; otelixizumab 9 mg, 0.80 ± 0.29; 18 mg, 0.67 ± 0.35; 27 mg, 0.60 ± 0.24). The number of participants meeting the definition of being a C-peptide responder at month 24 was: placebo, 1 (20%); otelixizumab 9 mg, 6 (67%); 18 mg, 2 (25%); 27 mg, 4 (57%).

Mean AUC glucose (mmol/l × min) during MMTT was below baseline only in the otelixizumab 9 mg group, at months 3, 6 and 24 (but not month 12 or 18) and was numerically lower than placebo at all postbaseline timepoints (ESM Fig. 1). In the placebo and otelixizumab 18 and 27 mg groups, change from baseline in mean AUC glucose was above baseline at all follow-up time points.

Following the hyperglycaemic clamp test, change from baseline in C-peptide and glucose weighted mean AUC_60–140 min_ showed a similar pattern of results to that of MMTT, in that the otelixizumab 9 mg dose showed the best treatment response compared with placebo (ESM Figs 2, 3).

Mean daily insulin use decreased in all groups from baseline to month 3, and then gradually increased over time in all groups with no statistically significant differences observed between the placebo and active-treatment groups. Change from baseline in HbA_1c_ levels (absolute and %) showed fluctuations over 24 months in all groups, and no marked differences between placebo and active treatment were observed (data not shown).

### Immunogenicity

All recorded participants who received otelixizumab showed positive anti-otelixizumab-binding antibody status at month 3 and remained positive at month 6. Two participants in the placebo group had positive anti-otelixizumab-binding results predose, which remained positive throughout.

### Exploratory biomarkers

#### T cell clonal expansion

**Fig. 4:**
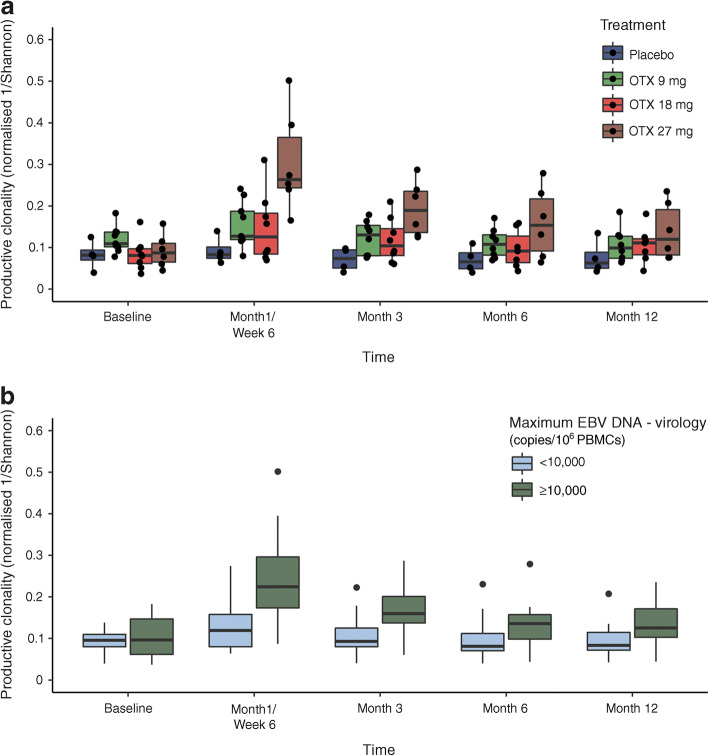
(**a**) Productive clonality over time by treatment group. (**b**) Box plot of productive clonality grouped by maximum EBV viral load. Boxes depict the IQR with lines for the 25th percentile, median and 75th percentile. The whiskers (vertical lines) denote the largest value within 1.5× IQR above 75th percentile or below the 25th percentile. Black circles denote individual participant data (**a**) and individual outliers (**b**). OTX, otelixizumab

Given that EBV clinical manifestations and elevated levels of EBV viral load were found to be transient, the use of TCR β chain immunosequencing to monitor T cell responses following EBV reactivation enabled us, without a technical bias towards known antigens, to determine whether T cells expressing shared TCR β chains expanded following EBV reactivation. Changes in productive clonality were consistently observed at all three otelixizumab dose levels; increases peaked at month 1/week 6 (Fig. 4a), in line with EBV viral load values returning to the reference range. At month 1/week 6, the mean ± SD change from baseline was 0.010 ± 0.012, 0.041 ± 0.057, 0.063 ± 0.084 and 0.318 ± 0.131 for the placebo and otelixizumab 9, 18 and 27 mg groups, respectively. The otelixizumab 27 mg group had statistically significant higher clonality compared with placebo at this timepoint (*p* = 0.009); however, neither otelixizumab 9 mg nor 18 mg reached statistical significance vs placebo (*p* = 0.075 and *p* = 0.28, respectively). These effects diminished over time; by month 6, productive clonality returned to baseline levels in participants on otelixizumab 9 mg (mean ± SD change from baseline −0.012 ± 0.035, *p* = 0.46), whereas levels in the 18 and 27 mg groups were still marginally elevated at month 12 compared with baseline (mean ± SD change from baseline 0.024 ± 0.0185, *p* = 0.02, and 0.034 ± 0.029, *p* = 0.06, respectively). Productive clonality in the placebo group remained relatively stable over time.

As the ‘synchronised’ kinetics with EBV viral load resolved within 2 months postdose and increased clonality peaked as early as 1 month/6 weeks post treatment, we investigated whether EBV antigens were the likely cause of the T cell expansion, rather than a non-specific agonistic signal delivered by otelixizumab following CD3ε target engagement. In a subgroup analysis, we found that participants experiencing EBV reactivation (maximum EBV viral load >10,000 copies/10^6^ PBMCs) mounted a stronger T cell response than those without EBV reactivation, as suggested by increased productive clonality (Fig. 4b).

A comparison of TCR β chain immunosequencing data queries against a databank holding sequences of EBV-reactive TCR clones from the reported literature, and limited flow cytometry analysis using HLA-A2 restricted multimers (ESM Fig. 4), provided further evidence that the observed increased clonality is related to EBV-specific T cell responses.

#### Lymphocyte subsets

**Fig. 5:**
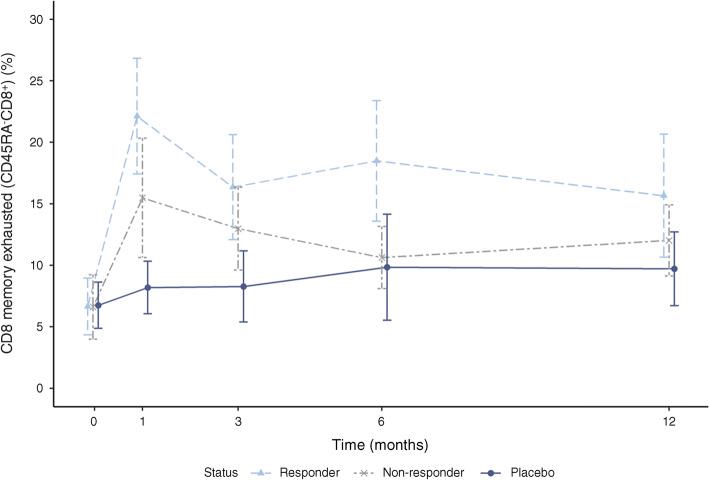
Mean ± SEM partially exhausted CD8^+^ memory T cells over time by metabolic response

Except for CD8^+^ T cells and some subsets thereof, there were no consistent otelixizumab-related changes from baseline in either the absolute numbers or percentages of any other cell lineages (ESM Table 4). Increases from baseline in partially exhausted memory CD8^+^ T cells, based on a CD3^+^CD8^+^CD45RA Eomes^+^KLRG1^+^TIGIT^+^ phenotype, were consistently observed in all three dose groups as early as month 1/week 6. Mean ± SD percentage of partially exhausted CD8^+^ memory T cells increased from 9.5 ± 10.21% at baseline to a peak of 28.5 ± 13.84% in the otelixizumab 9 mg group, from 6.5 ± 6.52% to a peak of 17.0 ± 16.35% in the otelixizumab 18 mg group, and from 2.7 ± 1.65% to a peak of 16.2 ± 13.23% in the otelixizumab 27 mg group. Increase from baseline in partially exhausted CD8^+^ memory T cells was more pronounced in participants on otelixizumab who were retrospectively defined as metabolic responders (Fig. 5).

## Discussion

In this Phase I/II study, which was primarily designed to evaluate the safety and tolerability of four cumulative doses of otelixizumab, three cumulative otelixizumab doses (9, 18 and 27 mg) were tested and only the 9 and 18 mg doses were well tolerated. The highest planned dose (36 mg) was not given because of predefined protocol stopping criteria on EBV reactivation being met with the 27 mg dose. Following administration of the three doses of otelixizumab, and consistent with findings from previous studies [9, 10, 23], dose-related CRS AEs and EBV reactivation were reported in this study, despite prophylaxis. During the dosing period, none of the most commonly reported AEs (headache, nausea, vomiting and rash) were above grade 3 toxicity, and nausea and vomiting occurred less frequently in the otelixizumab 9 mg group than in the 18 mg and 27 mg groups, albeit more frequently than with placebo. In comparison, previous studies have reported that 3 weeks after i.v. administration of otelixizumab 48 mg, 75% of participants developed a sore throat and 25%, developed fever and cervical adenopathy, both of which were transient and related to EBV reactivation [9, 23]; these symptoms were not reported after administration of otelixizumab 3.1 mg [16, 17].

In our study, EBV reactivation was also dose related; no participants in the otelixizumab 9 mg group had any clinical sign of EBV reactivation, although there was evidence of an increased EBV viral load in some but not all participants in this dosing group. Although EBV reactivation is indicative of a compromised immune system, in agreement with observations made by Keymeulen and colleagues [23], immunocompetence appears to be rapidly restored as evidenced by an expansion of T cells within 6 weeks postdosing, preceding long-term control over EBV reactivation. In a recent study of individuals at high risk of developing type 1 diabetes, teplizumab at an exposure equivalent to a total cumulative dose of otelixizumab 9 mg resulted in only one participant having EBV-related symptoms (pharyngitis, rhinorrhoea and cough) among eight participants with quantifiable EBV DNA in whole blood at weeks 3 and 6 after dosing [24]. Thus, otelixizumab appears to have a similar profile to teplizumab in terms of EBV reactivation, and possibly a slightly worse profile with respect to CRS symptoms. Given the small number of participants who received the 9 mg otelixizumab dose and limited published safety data on an exposure-equivalent teplizumab dose, caution may be warranted in interpreting these findings.

During both the present study and in a previous Belgian Diabetes Registry study [23], no negative long-term effects of treatment were reported, suggesting that EBV reactivation after otelixizumab administration is rather benign and, above all, resolves within a short time frame.

This study, however, has some limitations. First, it was not designed or powered to answer the question of whether doses of otelixizumab from 9 to 36 mg are associated with better or more prolonged maintenance of beta cell function. Second, additional confounding factors have been identified, namely the higher mean baseline beta cell function observed in the otelixizumab 9 mg group and the implementation of a protocol amendment that permitted the additional prophylactic medication ondansetron, a 5HT_3_-receptor antagonist, in participants receiving otelixizumab 18 mg or more and the effects of which are unknown. Third, this study did not allow us to thoroughly examine the relationship between residual beta cell function and insulin requirements, as recording of insulin use was limited to 7 days before each visit. Finally, good-quality PBMCs for flow analysis were not consistently obtained and multimer analysis was restricted to those who were HLA-A2-positive, thus reducing the number of evaluable participants for immune monitoring.

Previous investigations of an otelixizumab 48 mg cumulative dose demonstrated that preservation of beta cell function depends not only on anti-CD3 therapy, but also on baseline participant characteristics such as younger age, higher baseline beta cell function and the presence of insulin autoantibodies [9, 11, 13]. Recently, teplizumab proved to be efficacious in delaying the onset of overt diabetes in a subgroup of beta cell autoantibody-positive individuals with dysglycaemia [24], providing evidence of the first immune intervention able to delay the diagnosis of diabetes and the requirement for insulin treatment. Our study showed preservation of beta cell function over 18 months in the otelixizumab 9 mg group but not in the 18 and 27 mg groups. The higher mean baseline beta cell function observed in the 9 mg group was not identified as a statistically significant covariate and could be an artefact of sample size, and thus a link between baseline beta cell function and treatment response cannot be ruled out. In this study it was not possible to stratify by baseline beta cell function because of the dose-escalation nature of the study design, but this is recommended in future trials in this disease where beta cell function is being assessed and is a primary endpoint.

In this trial, increases from baseline in partially exhausted CD8^+^ memory T cells were more pronounced in participants dosed with otelixizumab who were retrospectively defined as metabolic responders. This is consistent with findings described for teplizumab [20]. However, our study established that partially exhausted memory CD8^+^ T cells can expand as early as 1 month after treatment, and this is the earliest time point any study has looked into detailed immunophenotyping. Although the specificity of the partially exhausted CD8^+^ memory T cells has thus far not been elucidated, it is likely that both autoreactive and viral-reactive T cells are contained within this population of T cells. Herold and colleagues recently hypothesised that following CD3ɛ target engagement by monoclonal antibodies, T cells with high avidity for viral antigens (such as those associated with EBV) may be affected differently to autoreactive T cells with lower avidity [24]. While currently the signatures identified through TCR β chain immunosequencing were dominated by EBV reactivation, as the field continues to map specific complementary determining region 3 (CDR3) sequences to their cognate antigens, these data may help to further characterise the T cells involved in pathogenesis and response to therapy.

In summary, in this dose-finding study, 18 mg was the maximum tolerated dose of otelixizumab and 9 mg was identified as the dose with the better therapeutic index, given the low incidence of transient symptoms related to CRS, the lack of clinical EBV reactivation and early changes in immunophenotyping such as expansion of exhausted memory CD8^+^ T cells, which was more pronounced in participants defined as metabolic responders. These observations might help to clarify the margins of safety and tolerability of anti-CD3 monoclonal antibody treatment, both in type 1 diabetes and in delaying the progression to overt type 1 diabetes.

## Supplementary Information

ESM (PDF 577 kb)

## Data Availability

Anonymised individual participant data and study documents can be requested for further research from www.ClinicalStudyDataRequest.com.

## Abbreviations

AE: Adverse event
CRS: Cytokine release syndrome
EBV: Epstein–Barr virus
MMTT: Mixed-meal tolerance test
PBMC: Peripheral blood mononuclear cell
SAE: Serious adverse event
TCR: T cell receptor
