# Supplementary material for: A randomised, single-blind, placebo-controlled, dose-finding safety and tolerability study of the anti-CD3 monoclonal antibody otelixizumab in new-onset type 1 diabetes
Source: Diabetologia. 2020 Nov 4;64(2):313–24. doi: 10.1007/s00125-020-05317-y (PMC7801303; doi:10.1007/s00125-020-05317-y)

Dose-finding safety and tolerability study of anti-CD3 monoclonal antibody otelixizumab in new onset Type 1 diabetes

Bart Keymeulen, André van Maurik, Dave Inman, João Oliveira, Rene McLaughlin, Rachel M Gittelman, Bart O. Roep, Pieter Gillard, Robert Hilbrands, Frans Gorus, Chantal Mathieu, Ursule Van de Velde, Nicolas Wisniacki, Antonella Napolitano

Electronic supplementary material

Abbreviations

CPT: Cell Preparation Tube

FDA: Food and Drug Administration

ULN: Upper limit of normal

ESM Methods

Safety

Liver chemistry stopping criteria

Liver chemistry threshold stopping criteria were designed to assure patient safety and to evaluate liver event etiology (in alignment with the FDA premarketing clinical liver safety guidance). Study treatment was stopped for a patient if any of the following liver chemistry stopping criteria were met:

1. ALT ≥ 3xULN and bilirubin ≥ 2xULN

   Serum bilirubin fractionation should have been performed if testing was available. If fractionation was unavailable, urinary bilirubin was to be measured via dipstick (a measurement of direct bilirubin, which would suggest liver injury).
2. ALT ≥ 5xULN.
3. ALT ≥ 3xULN if associated with symptoms (new or worsening) believed to be related to hepatitis (such as fatigue, nausea, vomiting, right upper quadrant pain or tenderness or jaundice) or believed to be related to hypersensitivity (such as fever, rash or eosinophilia).
4. ALT ≥ 3xULN persists for ≥ 4 weeks.
5. ALT ≥ 3xULN and cannot be monitored weekly for 4 weeks.

Subjects with ALT ≥ 3xULN and <5xULN and bilirubin <2xULN, who did not exhibit hepatitis symptoms or rash, could continue study treatment as long as they could be monitored weekly for 4 weeks.

Exploratory biomarkers

Blood samples for analysis of exploratory biomarkers were collected at pre-dose baseline, pre-dose on day 6 of dosing, at week 6, and at months 3, 6 and 24. Blood was collected into sodium citrate CPT™ tubes (BD Biosciences) and PBMC fractions were separated and cryopreserved at the clinical sites, within 8 hours of collection to stabilise the samples.

For enumeration of lymphocyte subsets, cryopreserved PBMCs were thawed, washed and stained with surface antibodies, including CD3, CD8, CD45RA, KLRG1 and TIGIT, before permeabilization (FOXP3 Fix/Perm Buffer Set, BioLegend) for EOMES staining, adapted from Long et al (1); defining CD3+CD8-EOMES+KLRG1+TIGIT+ as a partially exhausted phenotype. In an additional flow cytometry panel, thawed PBMCs were stained to identify T cell subsets (Th1, Th2, Th17, Tc1, Tc2, Tc17, CD4 Treg and CD8 Treg) using surface antibodies: CD4, CD8, CD45RA, CD25, CD127, CXCR3 and CCR6. To allow subject stratification for further testing (i.e. quantification of EBV-reactive CD8 T cells, restricted to HLA-A2 positive subjects), HLA-A2 surface staining was included. Data were acquired on a FACS Canto II using FACS Diva Software and analysed using FlowJo X. Isotype controls were used to define negative population - positive gates were set to include ~1% of events in these samples.

The identification and quantification of HLA-A2 restricted EBV-reactive CD8 T cells (Latent Membrane Protein-2 426-434 CLGGLLTMV) from cryopreserved PBMCs was performed using the Diab-Q-kit, developed by Leiden University Medical Center (2). The Diab-Q-kit uses the combinatorial encoding method whereby epitope specific CD8 T cells are identified by staining with a combination of two HLA class I multimers that have the same epitope but different fluorochromes. Data were acquired and analysed on a BD LSRII using FACS Diva Software.

Immunosequencing of the CDR3 regions of human TCRβ chains was performed using the ImmunoSEQ™ Assay (Adaptive Biotechnologies, Seattle, WA). For the preparation of cDNA, RNA was isolated from PAXgene Blood by Q^2 Solutions using Thermo-Fisher High Capacity RNA-to-cDNA kit. cDNA was amplified in a bias-controlled multiplex PCR, followed by high-throughput sequencing. Sequences were collapsed and filtered in order to identify and quantitate the absolute abundance of each unique TCRβ CDR3 region for further analysis as previously described (3-5). Productive clonality was used to assess the degree of mono or oligoclonality within the TCRβ repertoire. Clonality was defined as 1- Peilou's

eveness (6), and was calculated on productive rearrangements by: $1+\frac{\sum_{i}^{N} p_i \log_2(p_i)}{\log_2(N)}$ where

pi is the proportional abundance of rearrangement i and N is the total number of rearrangements. The productive clonality statistic was used to measure skew in the T-cell repertoire towards high frequency, expanded T-cell clones. Productive clonality values range from 0 to 1 and describe the shape of the frequency distribution: values approaching 1 indicate an increasingly asymmetric distribution in which a few clones are present at high frequencies. Statistical analysis was performed in R version 3.2.

Sequence and meta data that have been made publicly available were used for querying CDR3 amino acid sequences from EBV-reactive TCRβ sequences against each repertoire and summed the frequencies of all matched clones on a per sample basis (7-26).

ESM References

1. Long, S.A. *et al*. Remodeling T cell compartments during anti-CD3 immunotherapy of type 1 diabetes. *Cell. Immunol*. **319**, 3-9 (2017).
2. Velthuis, J.H. *et al*. Simultaneous detection of circulating autoreactive CD8+ T-cells specific for different islet cell-associated epitopes using combinatorial MHC multimers. *Diabetes*. 59, 1721-30 (2010).
3. Robins, H.S. *et al*. Comprehensive assessment of T-cell receptor β-chain diversity in αβ T cells. *Blood.* **114**, 4099-4107 (2009).
4. Carlson, C.S. *et al*. Using synthetic templates to design an unbiased multiplex PCR assay. *Nat. Commun.* **4**, 2680 (2013).
5. Robins, H.S. *et al*. Ultra-sensitive detection of rare T cell clones. *J. Immunol. Methods.* **375**, 14-9 (2012).
6. Kirsch, I., Vignali, M., Robins, H. T-cell Receptor profiling in cancer. *Mol. Oncol.* **9**, 2063-70 (2015).
7. Silins, S.L. et al. Development of Epstein-Barr virus-specific memory T cell receptor clonotypes in acute infectious mononucleosis. *J Exp Med*. 1996 **184,** 1815-24 (1996).
8. Silins, S.L. et al. Selection of a diverse TCR repertoire in response to an Epstein-Barr virus-encoded transactivator protein BZLF1 by CD8+ cytotoxic T lymphocytes during primary and persistent infection. *Int Immunol.* **9**, 1745-55 (1997).
9. Silins, S.L., Cross, S.M., Krauer, K.G., Moss, D.J., Schmidt, C.W., Misko, I.S. A functional link for major TCR expansions in healthy adults caused by persistent Epstein-Barr virus infection. *J Clin Invest*. **102**, 1551-8 (1998).
10. Burrows, S.R., Silins, S.L., Moss, D.J., Khanna, R., Misko, I.S., Argaet, V.P. T cell receptor repertoire for a viral epitope in humans is diversified by tolerance to a background major histocompatibility complex antigen. *J Exp Med*. **182,** 1703-15 (1995).

11. Klarenbeek, P.L. et al. Deep sequencing of antiviral T-cell responses to HCMV and EBV in humans reveals a stable repertoire that is maintained for many years. *PLoS Pathog*. **8,** e1002889 (2012).
12. Lim, A. et al. Frequent contribution of T cell clonotypes with public TCR features to the chronic response against a dominant EBV-derived epitope: application to direct detection of their molecular imprint on the human peripheral T cell repertoire. *J Immunol*. **165,** 2001-11 (2000).
13. Miles, J.J. et al. Genetic and Structural Basis for Selection of a Ubiquitous T Cell Receptor Deployed in Epstein-Barr Virus Infection. *PLoS Pathog*. **6,** e1001198 (2010).
14. Annels, N.E., Callan, M.F.C, Tan, L., Rickinson, A.B. Changing Patterns of Dominant TCR Usage with Maturation of an EBV-Specific Cytotoxic T Cell Response. *J Immunol*. *165*, 4831-4841 (2000).
15. Silins, S.L. et al. Asymptomatic primary Epstein-Barr virus infection occurs in the absence of blood T-cell repertoire perturbations despite high levels of systemic viral load. *Blood*. **98,** 3739-44 (2001).
16. Couedel, C., Bodinier, M., Peyrat, M.A., Bonneville, M., Davodeau, F., Lang, F. Selection and long-term persistence of reactive CTL clones during an EBV chronic response are determined by avidity, CD8 variable contribution compensating for differences in TCR affinities. *J Immunol*. **162,** 6351-8 (1999).
17. Miconnet, I. et al. Large TCR diversity of virus-specific CD8 T cells provides the mechanistic basis for massive TCR renewal after antigen exposure. *J Immunol*. **186**, 7039-49 (2011).
18. Levitsky, V., de Campos-Lima, P.O., Frisan, T., Masucci, M.G. The clonal composition of a peptide-specific oligoclonal CTL repertoire selected in response to persistent EBV infection is stable over time. *J Immunol*. **161**, 594-601 (1998).
19. Miles, J.J. et al. TCR alpha genes direct MHC restriction in the potent human T cell response to a class I-bound viral epitope. *J Immunol*. **177**, 6804-14 (2006).

20. Miles, J.J. et al. CTL recognition of a bulged viral peptide involves biased TCR selection. *J Immunol*. **175**, 3826-34 (2005).
21. Neller, M.A., Burrows, J.M., Rist, M.J., Miles, J.J., Burrows, S.R. J Virol. High frequency of herpesvirus-specific clonotypes in the human T cell repertoire can remain stable over decades with minimal turnover. *J Virol*. **87**, 697-700 (2013).
22. Iancu, E.M. et al. Persistence of EBV antigen-specific CD8 T cell clonotypes during homeostatic immune reconstitution in cancer patients. *PLoS One*. **8**, e78686 (2013).
23. Koning, D. et al. CD8+ TCR repertoire formation is guided primarily by the peptide component of the antigenic complex. *J Immunol*. **190**, 931-9 (2013).
24. Venturi, V. et al. TCR beta-chain sharing in human CD8+ T cell responses to cytomegalovirus and EBV. *J Immunol*. **181**, 7853-62 (2008).
25. Clute, S.C. et al. Broad cross-reactive TCR repertoires recognizing dissimilar Epstein-Barr and influenza A virus epitopes. *J Immunol*. **185**, 6753-64 (2010).
26. Iancu, E.M. et al. Clonotype selection and composition of human CD8 T cells specific for persistent herpes viruses varies with differentiation but is stable over time. *J Immunol*. **183**, 319-31 (2009).

ESM Table 1: Dosage cohorts

Cohort	Cumulative dose (mg)	Day 1			Day 2			Day 3			Days 4-6		
		Daily dose (mg)	Rate of infusion (mg/h)	Infusion time (h)	Daily dose (mg)	Rate of infusion (mg/h)	Infusion time (h)	Daily dose (mg)	Rate of infusion (mg/h)	Infusion time (h)	Daily dose (mg)	Rate of infusion (mg/h)	Infusion time (h)
**1**	9	1.5	0.167	9	1.5	0.25	6	1.5	0.5	3	1.5	1.5	1
**2**	18	3	0.25	12	3	0.5	6	3	1	3	3	3	1
**3**	27	4.5	0.375	12	4.5	0.75	6	4.5	1.5	3	4.5	4.5	1

ESM Table 2: CRS AE Grading System

Adverse event	Grade 1	Grade 2	Grade 3	Grade 4	Grade 5
Headache	Mild pain	Moderate pain; limiting instrumental ADL	Severe pain: limiting self-care ADL	-	-
Fever	38.0-39.0°C	>39.0-40.0°C	>40.0°C for ≤ 24 hrs	>40.0°C for > 24 hrs	Death
Vomiting	1-2 episodes (separated by 5 minutes) in 24 hrs	3-5 episodes (separated by 5 minutes) in 24 hrs	≥6 episodes (separated by 5 minutes) in 24 hrs	Life-threatening consequences; urgent intervention indicated	Death
Diarrhoea	Increase of <4 stools per day over baseline	Increase of 4-6 stools per day over baseline	Increase of ≥7 stools per day over baseline; incontinence; limiting self-care ADL	Life-threatening consequences; urgent intervention indicated	Death
Hypotension	Asymptomatic, intervention not indicated	Non-urgent medical intervention indicated	Urgent medical intervention indicated	Life-threatening consequences; urgent intervention indicated	Death
Chills	Mild sensation of cold; shivering; chattering of teeth	Moderate tremor of the entire body; narcotics indicated	Severe or prolonged; not responsive to narcotics	-	-
Nausea	Loss of appetite without alteration in eating habits	Oral intake decreased without significant weight loss, dehydration or malnutrition	Inadequate oral caloric or fluid intake	-	-
Arthralgia	Mild pain	Moderate pain; limiting instrumental ADL	Severe pain: limiting self-care ADL	-	-
Myalgia	Mild pain	Moderate pain; limiting instrumental ADL	Severe pain: limiting self-care ADL	-	-

ADL: Activities of daily living

ESM Table 3: Summary of drug-related adverse events by maximum toxicity grade*, n (%)

	Placebo (N=5)	OTX 9 mg (N=9)	OTX18 mg (N=8)	OTX 27 mg (N=7)
**Overall**				
Any Grade 1 to 5 drug-related event**	5 (100)	9 (100)	8 (100)	7 (100)
Grade 1	3 (60)	1 (11)	3 (38)	2 (29)
Grade 2	2 (40)	4 (44)	2 (25)	3 (43)
Grade 3	0	4 (44)	3 (38)	2 (29)
**Dosing period**				
Any Grade 1 to 5 drug-related event**	4 (80)	9 (100)	8 (100)	7 (100)
Grade 1	3 (6)	1 (11)	4 (50)	2 (29)
Grade 2	1 (20)	4 (44)	1 (13)	3 (43)
Grade 3	0	4 (44)	3 (38)	2 (29)
**Post-dose to week 6**				
Any Grade 1 to 5 drug-related event**	3 (60)	7 (78)	7 (88)	7 (100)
Grade 1	2 (40)	7 (78)	6 (75)	4 (57)
Grade 2	1 (20)	0	1 (13)	3 (43)
Grade 3	0	0	0	0
**Week 6 to month 24**				
Any Grade 1 to 5 drug-related event**	5 (100)	8 (89)	5 (63)	5 (71)
Grade 1	5 (100)	8 (89)	5 (63)	4 (57)
Grade 2	0	0	0	1 (14)
Grade 3	0	0	0	0

*Definition of grades: See ESM Table 2
**There were no Grade 4 or 5 events reported

ESM Table 4: Lymphocyte subsets: Mean (Standard Error) change from baseline at month 1/week 6.

Parameter	Placebo	OTX 9 mg	OTX 18 mg	OTX 27 mg
CD3 (10^9/L)<br>T Lymphocytes	-0.60 (0.408)	0.055 (0.1141)	-0.14 (0.235)	0.98 (0.303)
CD3/Lymphocytes (%)<br>T Lymphocytes	-1.75 (1.109)	-1.33 (1.498)	0.000 (1.2536)	4.67 (1.498)
CD4 (10^6/L)<br>Th cells	-318 (186.4)	-198 (36.9)	-224 (124.8)	-95.0 (65.36)
CD4/Lymphocytes (%)<br>Th cells	-2.50 (1.708)	-9.33 (1.856)	-5.71 (1.755)	-18.3 (2.29)
CD4+CD25+CD127- (10^6/L)<br>CD4 Treg	-8.02 (7.696)	-4.19 (5.024)	-23.9 (8.44)	3.44 (5.286)
CD4+CD25+CD127-/CD4+ (%)<br>CD4 Treg	0.39 (0.555)	0.42 (0.949)	-1.29 (0.602)	1.08 (0.798)
CD4+CXCR3+CCR6- (10^6/L)<br>Th1	-169 (97.1)	-27.2 (43.39)	-48.2 (25.46)	42.4 (28.79)
CD4+CXCR3+CCR6-/CD4+ (%)<br>Th1	-3.26 (6.048)	-1.03 (3.764)	0.62 (3.018)	8.47 (3.232)
CD4+CXCR3-CCR6- (10^6/L)<br>Th2	-322 (389.9)	-215 (63.8)	-71.0 (157.40)	-76.4 (58.24)
CD4+CXCR3-CCR6-/CD4+ (%)<br>Th2	-9.52 (15.874)	-3.61 (6.437)	4.90 (7.324)	-8.56 (7.949)

Parameter	Placebo	OTX 9 mg	OTX 18 mg	OTX 27 mg
CD4+CXCR3-CCR6+ (10^6/L)<br>Th17	-37.5 (29.61)	4.65 (27.810)	-33.6 (22.25)	-6.35 (19.133)
CD4+CXCR3-CCR6+/CD4+ (%)<br>Th17	-1.78 (1.541)	1.87 (1.574)	-1.31 (2.148)	0.88 (1.994)
CD8 (10^6/L)<br>Tc	-105 (81.1)	290 (152.7)	136 (134.5)	1155 (249.3)
CD8/Lymphocytes (%)<br>Tc	1.00 (1.291)	10.0 (3.75)	7.57 (2.359)	24.0 (1.98)
CD8+CD25+CD127- (10^6/L)<br>CD8 Treg	3.63 (3.790)	0.69 (1.190)	1.35 (0.544)	24.7 (14.01)
CD8+CD25+CD127-/CD8+ (%)<br>CD8 Treg	0.37 (0.429)	-0.050 (0.1053)	0.082 (0.0289)	0.42 (0.394)
CD8+CXCR3+CCR6- (10^6/L)<br>Tc1	-171 (121.5)	104 (97.5)	187 (66.0)	755 (215.8)
CD8+CXCR3+CCR6-/CD8+ (%)<br>Tc1	-11.8 (9.46)	4.21 (9.394)	11.9 (2.94)	15.5 (5.28)
CD8+CXCR3-CCR6- (10^6/L)<br>Tc2	-90.4 (117.18)	5.14 (160.167)	-15.9 (94.28)	211 (80.2)
CD8+CXCR3-CCR6-/CD8+ (%)<br>Tc2	-4.54 (9.250)	-6.05 (10.961)	-10.4 (3.44)	-16.0 (5.71)
CD8+CXCR3-CCR6+ (10^6/L)<br>Tc17	0.94 (1.929)	31.8 (27.88)	-4.93 (2.444)	8.40 (5.533)

Parameter	Placebo	OTX 9 mg	OTX 18 mg	OTX 27 mg
CD8+CXCR3-CCR6+/CD8+ (%)<br>Tc17	-0.22 (0.547)	2.88 (1.301)	-0.82 (0.309)	-0.63 (0.382)

ESM Figure 1: Glucose weighted mean AUC 0-120 mins post MMTT

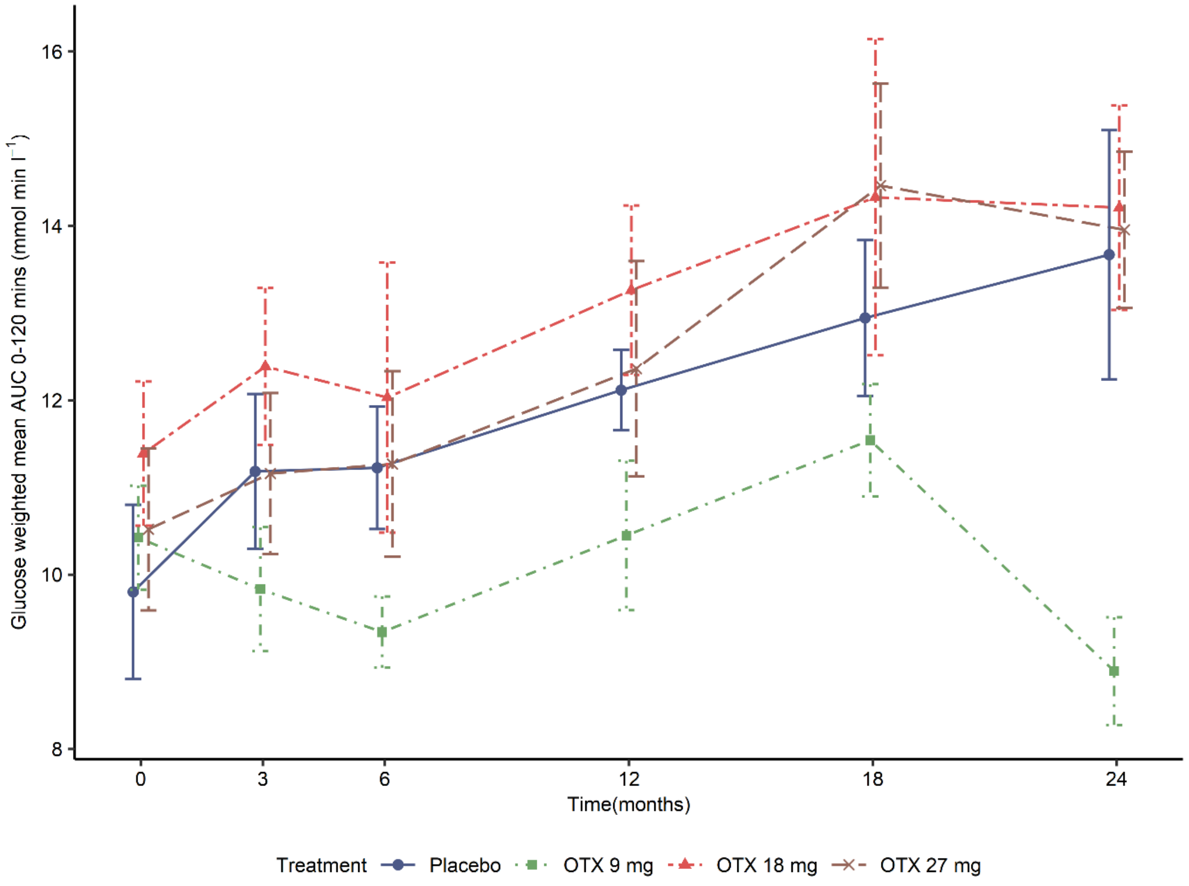

AUC: area under the curve; MMTT: Mixed-meal tolerance test; OTX: Otelixizumab

ESM Figure 2: C-peptide weighted mean AUC 60-140 mins post hyperglycaemic clamp test

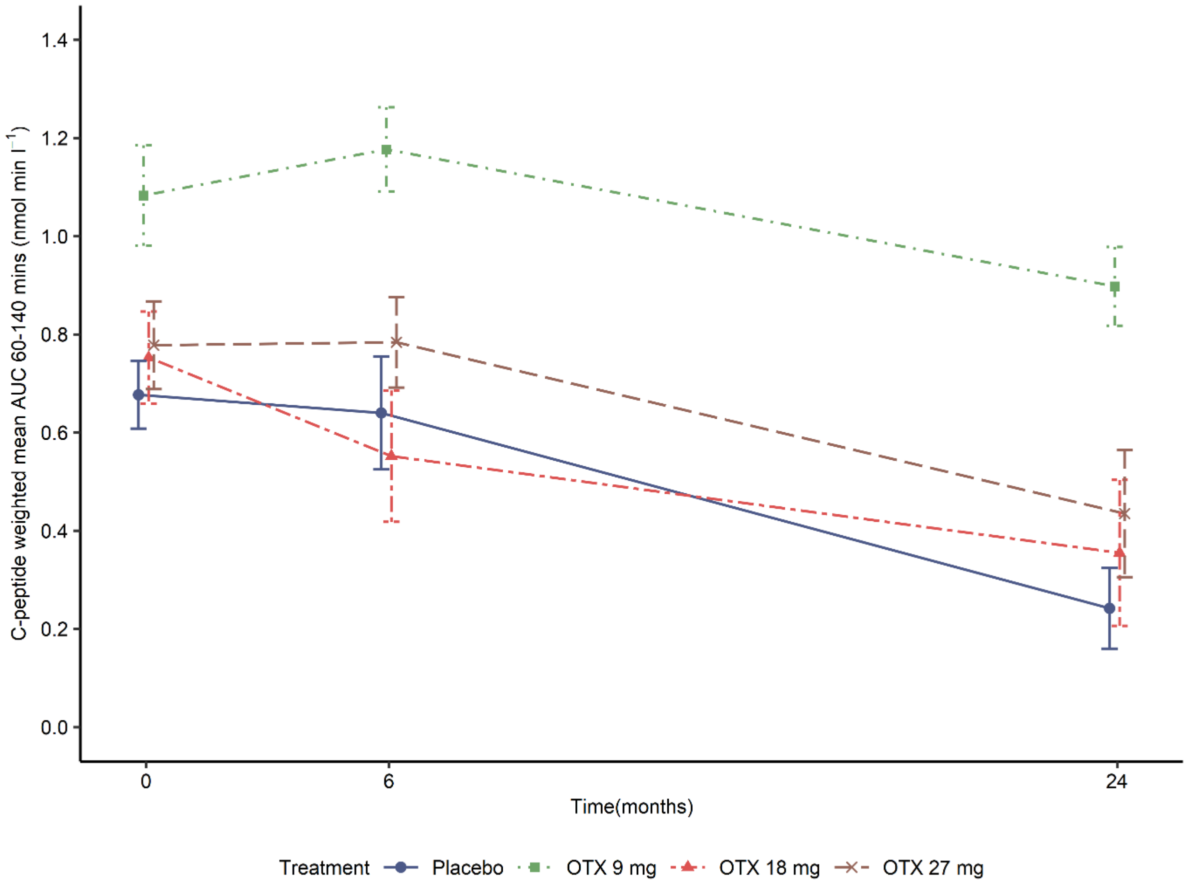

AUC: area under the curve; OTX: Otelixizumab

ESM Figure 3: Glucose weighted mean AUC 60-140 mins post hyperglycaemic clamp test

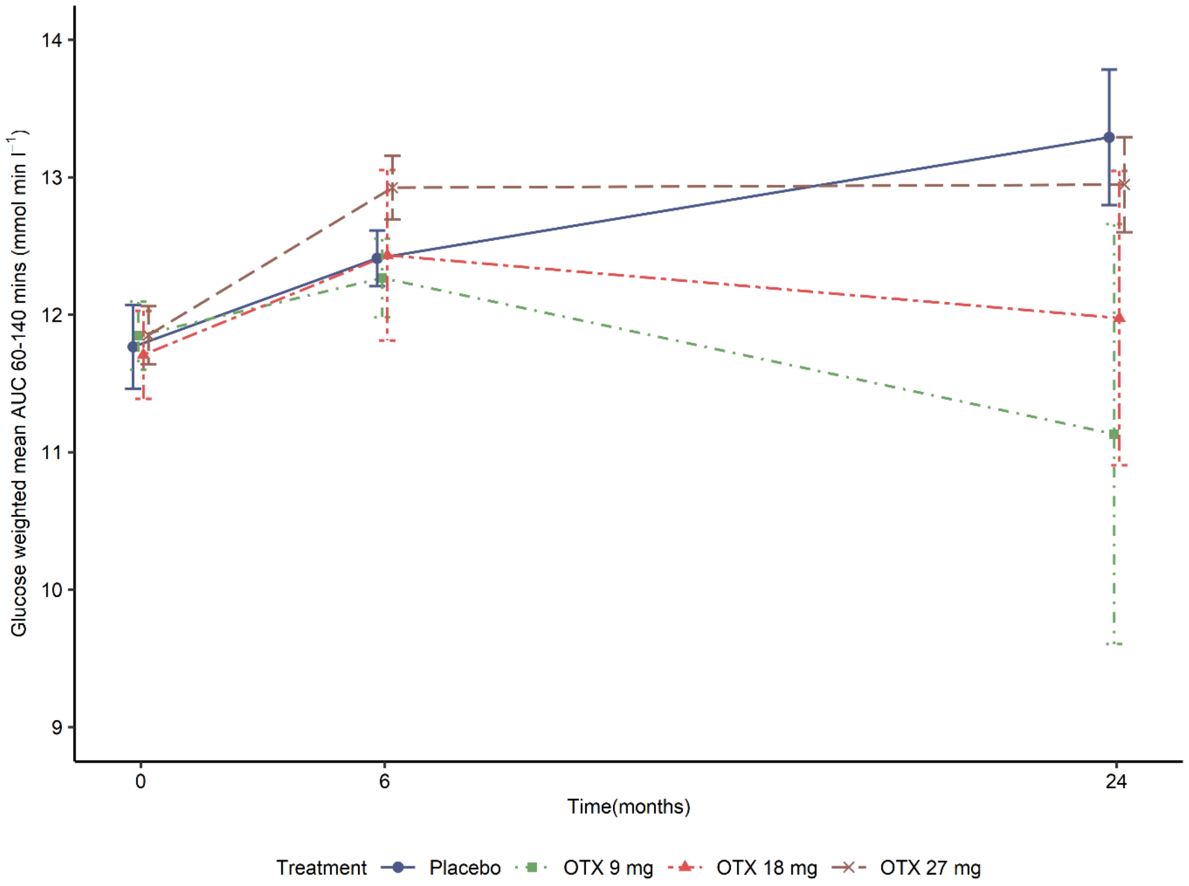

AUC: area under the curve; OTX: Otelixizumab

ESM Figure 4: Example of EBV-reactive CD8+ T-cell frequencies by multimer flow analysis (placebo vs 27 mg)

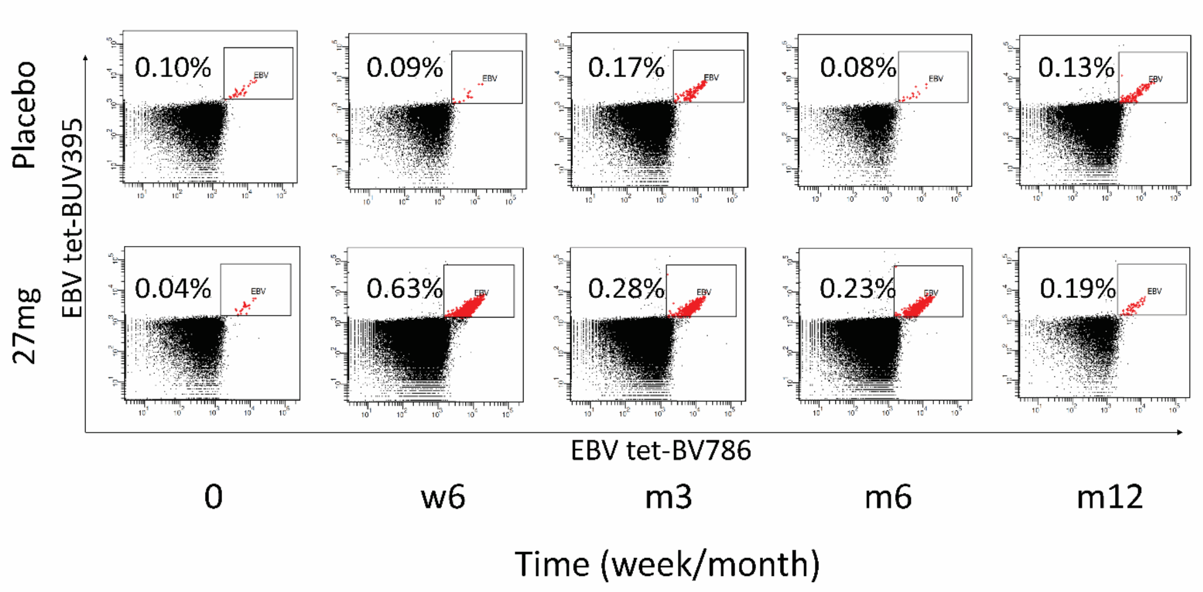

Supplement: Supplementary file 1 — (PDF 577 kb) [file 125_2020_5317_MOESM1_ESM.pdf]
